# Exploring Tantalum as a Potential Dopant to Promote the Thermoelectric Performance of Zinc Oxide

**DOI:** 10.3390/ma12132057

**Published:** 2019-06-26

**Authors:** Blanca I. Arias-Serrano, Wenjie Xie, Myriam H. Aguirre, David M. Tobaldi, Artur R. Sarabando, Shahed Rasekh, Sergey M. Mikhalev, Jorge R. Frade, Anke Weidenkaff, Andrei V. Kovalevsky

**Affiliations:** 1 CICECO-Aveiro Institute of Materials, Department of Materials and Ceramic Engineering, University of Aveiro, 3810-193 Aveiro, Portugal; 2 Materials and Resources, Techn, Universität Darmstadt, Alarich-Weiss Str.2, DE-64287 Darmstadt, Germany; 3 Condensed Matter Physics Department, University of Zaragoza and Institute of Material Science of Aragón, ICMA-CSIC, E-50018 Zaragoza, Spain; 4 Advanced Microscopy Laboratory, I+D Building-Campus Río Ebro, C/Mariano Esquillor s/n, 50018 Zaragoza, Spain; 5 i3N, Physics Department, University of Aveiro, 3810-193 Aveiro, Portugal; 6 TEMA-NRD, Mechanical Engineering Department, Aveiro Institute of Nanotechnology (AIN), University of Aveiro, 3810-193 Aveiro, Portugal

**Keywords:** zinc oxide, thermoelectrics, thermoelectric properties, donor doping, n-type semiconductor

## Abstract

Zinc oxide (ZnO) has being recognised as a potentially interesting thermoelectric material, allowing flexible tuning of the electrical properties by donor doping. This work focuses on the assessment of tantalum doping effects on the relevant structural, microstructural, optical and thermoelectric properties of ZnO. Processing of the samples with a nominal composition Zn_1−*x*_Ta*_x_*O by conventional solid-state route results in limited solubility of Ta in the wurtzite structure. Electronic doping is accompanied by the formation of other defects and dislocations as a compensation mechanism and simultaneous segregation of ZnTa_2_O_6_ at the grain boundaries. Highly defective structure and partial blocking of the grain boundaries suppress the electrical transport, while the evolution of Seebeck coefficient and band gap suggest that the charge carrier concentration continuously increases from *x* = 0 to 0.008. Thermal conductivity is almost not affected by the tantalum content. The highest *ZT*~0.07 at 1175 K observed for Zn_0.998_Ta_0.002_O is mainly provided by high Seebeck coefficient (−464 μV/K) along with a moderate electrical conductivity of ~13 S/cm. The results suggest that tantalum may represent a suitable dopant for thermoelectric zinc oxide, but this requires the application of specific processing methods and compositional design to enhance the solubility of Ta in wurtzite lattice.

## 1. Introduction

Design and development of thermoelectrics for high-temperature applications imposes several essential requirements on the material properties, with emphasis on high resistance against oxidation and thermodynamic stability under operation conditions. As compared to the traditional low-temperature thermoelectrics [1,2,3,4], oxides represent an appropriate family of materials and offer additional advantages such as relatively low toxicity and high natural abundance of the constituent elements [5]. Zinc oxide (ZnO) is an abundant wide band gap semiconductor possessing a variety of promising catalytic, optoelectronic and photochemical properties [6]. It was also considered as a potential thermoelectric material for high-temperature heat-to-electricity conversion [7,8], mainly due to the fact that the electronic transport properties can be flexibly tuned via donor doping, while excessively high thermal conductivity still remains a problem. 

Undoped ZnO possesses intrinsically low charge carrier concentration, insufficient for thermoelectric applications. Donor doping in zinc oxides can be achieved using elements with the oxidation state 3+ and appropriate coordination preferences to fit the ZnO wurtzite lattice. The most typical and commonly used dopant to explore various material design concepts is aluminium [7,8,9]; other representative examples include indium, iron, bismuth, nickel [10,11,12,13], etc. A promising strategy includes co-doping with various cations, in some cases leading to synergistically enhanced performance due to combined effects on the solubility in wurtzite lattice and microstructural evolution [14,15,16,17,18]. Doping with higher valence cations was also attempted, relying on more efficient electron doping effects due to the charge difference with Zn^2+^ cation [16,17,19]. 

This work explores the prospects for using tantalum as a dopant to promote the thermoelectric properties in zinc oxide ceramics. Up to now, the doping with tantalum was shown to be promising to enhance relevant optical properties and photocatalytic activity of ZnO-based thin films and nanoparticles [20,21,22,23,24,25]. Although the used processing and synthesis procedures apparently allow to achieve quite significant doping level for ZnO (even several at.% of tantalum), the studies show that only a part of the introduced tantalum acts as an efficient electronic donor [24]. Consolidation of highly-doped nanoparticles into ceramics, suitable as thermoelectric elements, requires elevated temperatures, which may significantly alter the doping level achieved in nanostructures. To our best knowledge, Zn(Ta)O was even not yet assessed as a potential thermoelectric material. Thus, in the present work, we relied on conventional solid-state route to produce the Ta-doped zinc oxide ceramics and to explore its relevant structural, microstructural and thermoelectric properties.

## 2. Materials and Methods

The samples with nominal composition Zn_1−*x*_Ta*_x_*O (*x* = 0, 0.002, 0.004, 0.006 and 0.008) were processed by solid-state method using ZnO (Alfa-Aesar, Haverhill, MA, USA, 99.99%) and Ta_2_O_5_ (Alfa Aesar, 99%) precursors. In accordance with the optimized procedure, the stoichiometric amounts of the precursor’s powders were first mixed in alcohol inside an ultrasonic bath, dried and annealed at 1223 K for 6 h three times with intermediate grindings. This was followed by ball-milling with ethanol, drying and preliminary uniaxial and final isostatic compacting at 200 MPa to produce green disk-shaped samples. Corresponding ceramic samples were sintered at 1773 K for 10 h in air. 

A part of sintered ceramics was grinded into the fine powder to perform X-ray diffraction (XRD) and diffuse reflectance spectroscopy (DRS) studies. Fractured and polished ceramic samples were used for microstructural studies by combined scanning electron microscopy/energy dispersive X-ray spectroscopy (SEM/EDS) techniques. In the case of polished samples an additional thermal etching step at 1573 K for 15 min was performed. The density (*ρ_exp_*) was calculated based on geometry and weight of the polished disc-shaped ceramic samples; at least three different samples for each composition were measured. For the combined total conductivity and Seebeck coefficient measurements the sintered ceramics were cut into rectangular bars ~1.5 × 2.5 × 15 mm^3^. Thermal diffusivity studies were performed on ~1.00 mm thick disc-shaped ceramic samples. For differential scanning calorimetry (DSC) measurement, small ceramic pieces with dimension of ~3 × 3 × 1 mm^3^ were used. 

The phase composition was examined by PANalytical X’Pert PRO diffractometer (Cu Kα) in the range 2θ = 20–80° (step of 0.02° and an exposition time of 200 s). Microstructural features of the ceramic samples were studied using SEM (Hitachi SU-70 instrument, Tokyo, Japan) and EDS (Bruker Quantax 400 detector, Billerica, MA, USA) equipment. Lamellaes for Transmission Electron Microscopy (TEM) analysis were prepared by Focused ion Beam by Dual Beam Helios Nanolab 650 from dense sintered pellets. Scanning Transmission Electron Microscopy with High Angular Annular Dark Field detector (STEM-HAADF) was used in a FEI Titan G2 TEM analysis. The optical band gap (*E_g_*) was assessed by DRS using a Shimadzu UV 3100 (JP) spectrometer, equipped with an integrating sphere and a white reference material, made of BaSO_4_ and Spectralon^®^, respectively. The spectra were acquired in the UV–Vis range (250–825 nm) with a step of 0.2 nm. Measurements techniques for the total electrical conductivity (*σ*) and Seebeck coefficient (*α*), and corresponding procedures for ZnO-based samples are described elsewhere [17,18,26]. Similar thermal equilibration profiles were used for the thermal diffusivity (*D*) and specific heat capacity (*C_p_*) studies (Netzsch LFA 457 Microflash and a Netzsch DSC 404F1 equipment, correspondingly). All electrical and thermal characterization was performed in air atmosphere.

## 3. Results

### 3.1. Evolution of Structural and Microstructural Properties

**Figure 1:**
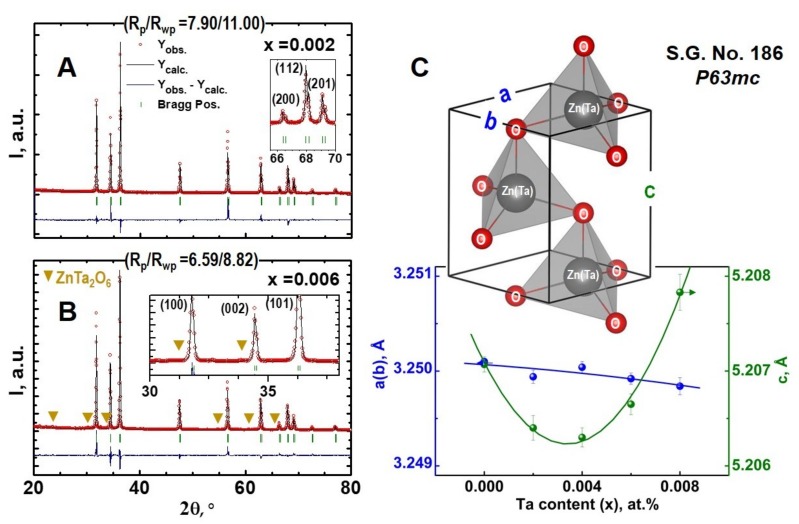
Room-temperature XRD patterns and profile fitting results for nominal Zn_0.998_Ta_0.002_O (**A**) and Zn_0.994_Ta_0.006_O (**B**) compositions; (**C**) wurtzite unit cell and composition dependence of the lattice parameters.

Representative XRD patterns of the prepared ceramic materials, showing the examples of fitting using a profile matching method in FullProf software [27] and corresponding parameters for the fit quality, are presented in Figure 1A,B. 

The XRD results suggest that the prepared materials are apparently single-phase at least up to *x* = 0.004 doping level. All the observed reflections can be indexed in a hexagonal wurtzite structure, space group (SG) P6_3_mc (Figure 1C), typical for ZnO-based ceramics processed under the described conditions. The samples *x* = 0.006 and 0.008 showed the presence of weak reflections marked by arrows in the inset in Figure 1B, which are likely to correspond to ZnTa_2_O_6_ phase, in agreement with the literature data [16,17,18]. The observed changes in the lattice parameters (Figure 1C), however, suggest that the tantalum addition also significantly affects the wurtzite unit cell size. The ionic radius of four-fold coordinated Zn^2+^ (0.60 Å) is essentially close to that of six-fold coordinated Ta^5+^ (0.64 Å) [28]; this difference is even expected to vanish on assuming a fraction of four-fold coordinated Ta^5+^. Thus, an “ideal” story for substitution of zinc for tantalum, illustrated in Figure 1C, when Ta^5+^ occupies the crystallographic positions of Zn^2+^, is not expected to result in significant variations of the lattice parameters. The literature data often suggests a contradictory behaviour of the *a* and *c* parameters on Ta doping in ZnO-based nanostructures and thin films [20,21,22,23,24,25]. The unit cell size can be significantly affected by the presence of additional defects in ZnO-based matrix, which, in turn, can be promoted both by the different dopant incorporation mechanisms and processing conditions (e.g., [6,29]). In fact, the latter justifies the above discrepancy in the literature data regarding the lattice parameters in thin films and nanoparticles. It is also believed that a softer bond between zinc and oxygen in the *c* direction makes this parameter more sensitive to the dopant content, in agreement with the XRD results obtained in this work (Figure 1C).

**Figure 2:**
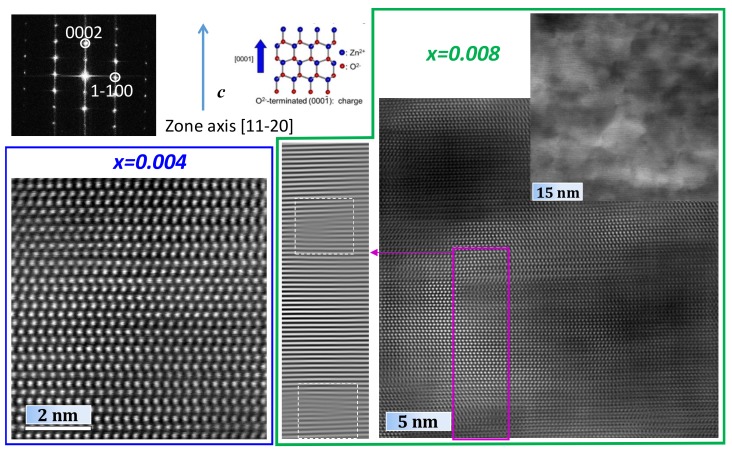
Selected area electron diffraction (SAED) pattern and HRSTEM-HAADF images obtained for *x* = 0.004 (blue line square) and 0.008 (edged by a thicker green line) samples. While sample with *x* = 0.004 shows homogeneous contrast, the contrast for *x* = 0.008 sample is rather inhomogeneous, with strain studies showing its origin in dislocations defects (left part edged by the green line).

Since the major part of the existing literature data on ZnO films and nanostructures still suggests a decrease in *c* parameter on tantalum doping, as observed in the sequence *x* = 0, 0.002, 0.004, further sudden increase of this parameter from *x* = 0.004 to 0.006 and 0.008 appears somewhat counterintuitive. The results of additional high-resolution STEM-HAADF studies with the corresponding strain analysis, performed for the samples *x* = 0.004 and 0.008, are shown in Figure 2.

**Figure 3:**
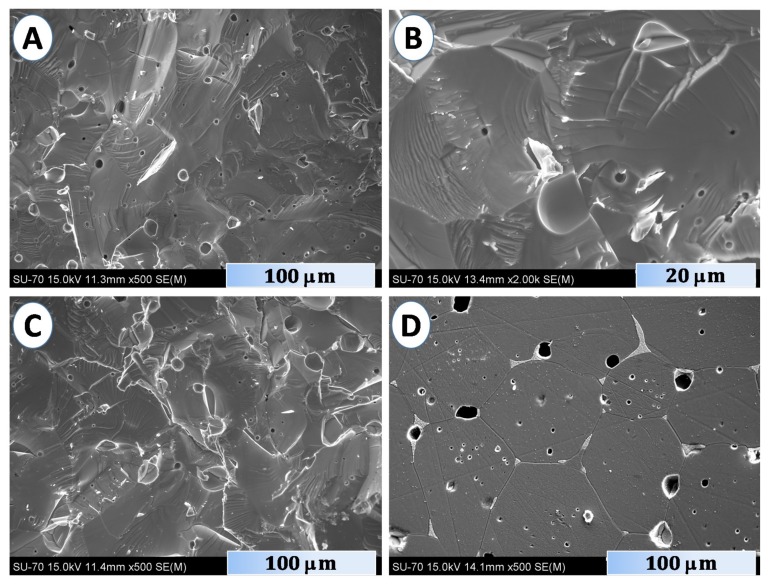
SEM microstructures of the fractured (**A**–**C**) and polished (**D**) ceramic samples with nominal Zn_1−x_Ta_x_O composition: *x* = 0.002 (**A**), *x* = 0.004 (**B**) and *x* = 0.008 (**C**,**D**).

These studies revealed a highly defective crystal structure in the case of Zn_0.992_Ta_0.008_O as compared to more “ideal” structure observed for Zn_0.996_Ta_0.004_O. In particular, massive formation of the dislocations can be responsible for the increase of the *c* parameter [6], observed for the samples with the highest nominal Ta content (Figure 2). Such dislocations are expected to compensate large stresses generated by the charge difference between Zn^2+^ and Ta^5+^ cations, thus allowing at least partial accommodation of the dopant in the wurtzite crystal lattice. At the same time, this does not result in the formation of macrodefects and appearance of excessive porosity, as indicated by the values of relative density, listed in the Table 1, and SEM micrographs, presented in Figure 3.

In fact, the porosity even decreases on increasing the tantalum content, while one may assume an opposite tendency based on presence of heavier tantalum in wurtzite lattice. It can hardly be assumed that this densification is promoted by traces of the ZnTa_2_O_6_ phase, whose theoretical density is roughly ~1.5 times higher than for wurtzite [30]. In any case, the relative densities in the range of 91 to 94% correspond to an acceptable level of the residual porosity, which is not expected to significantly affect the transport properties. High quality of the sintered ceramics is also confirmed by the SEM micrographs shown in Figure 3. The grain size of the wurtzite phase is relatively large (>50 μm) and does not show significant variations with the Ta content. 

**Figure 4:**
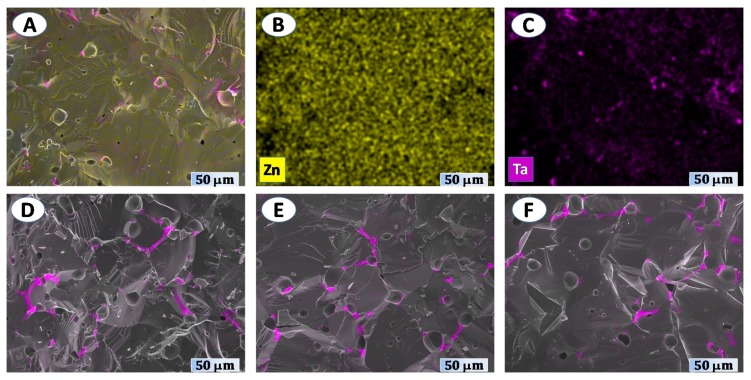
Energy-dispersive X-ray spectroscopy (EDS) mapping results for *x* = 0.02 (**A**–**C**), *x* = 0.04 (**D**), *x* = 0.06 (**E**) and *x* = 0.08 (**F**).

While the wurtzite matrix is represented by darker grains, better visible in Figure 3D, the presence of a distinct lighter phase at the grain boundaries is also very noticeable. This corresponds to the Ta-rich inclusions, most likely to the ZnTa_2_O_6_ phase. More guidelines regarding the spatial distribution of this phase can be drawn from the results of combined SEM/EDS analysis (Figure 4). 

The Ta-rich spots are observed even in the case of Zn_0.998_Ta_0.002_O sample (Figure 4A–C), suggesting that the actual doping level might be noticeably below that according to the nominal composition. This phase was not observed by the XRD studies for both *x* = 0.002 and *x* = 0.004 samples, likely due to vestigial low content. This apparent discrepancy between microstructural studies and XRD data is quite typical for ZnO-based materials, where the maximum doping level is limited by the electronic, coordination and steric preferences of zinc cations (e.g., [17]). For *x* = 0.002, ZnTa_2_O_6_ is presented mostly as localized spots, while for *x* ≥ 0.004 a partial blocking of the grain boundaries might be expected (Figure 4D–F), with corresponding effects on the electronic transport.

### 3.2. Electronic Transport Properties and Optical Band Gap

**Figure 5:**
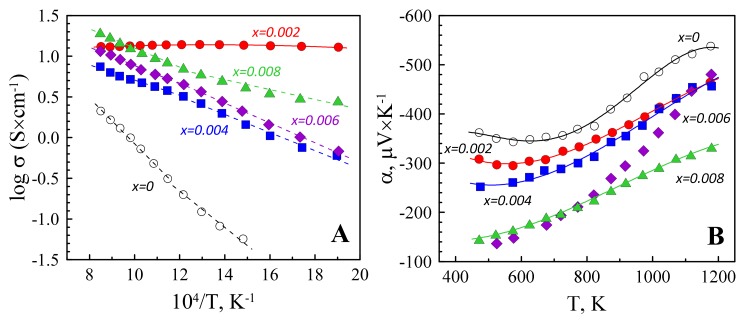
Temperature dependence of the electrical conductivity (**A**) and Seebeck coefficient (**B**) of Zn_1−*x*_Ta*_x_*O.

The above observed structural and microstructural features are in a good agreement with the obtained results on electrical properties (Figure 5).

Most of the samples demonstrate the temperature-activated behaviour of the electrical conductivity; therefore, Arrhenius-type representation of these data was selected. The corresponding activation energies are listed in the Table 1. The Zn_0.998_Ta_0.002_O is the only material showing a weak temperature dependence of the electrical conductivity, accompanied with a small *σ* decrease at T > 770 K, a behaviour similar to a degenerate semiconductor, desirable for a good thermoelectric material. At low and intermediate temperatures, the electrical conductivity of Zn_0.998_Ta_0.002_O is more than one order of magnitude higher than of pristine ZnO, processed under the identical conditions (Figure 5A). This unambiguously indicates the donor substitution in wurtzite lattice, corresponding values of the Seebeck coefficient are negative (Figure 5B), confirming expected n-type semiconducting material. It should be noticed that the pristine ZnO with a wurtzite structure is a natural n-type semiconductor, provided by the deviations from stoichiometry and presence of intrinsic defects like oxygen vacancies and zinc interstitials [6]. The nonlinear log *σ* vs. *T^−1^* behaviour was also observed for other donor-doped ZnO-based materials [31,32], and attributed to the temperature dependence of the contributions provided by the thermal excitation of electrons from donor levels to the conduction band, hopping between the impurity bands, oxygen vacancies, zinc interstitials and oxygen sorption–desorption processes to the overall charge transport mechanism. The activation energies for both high and low-temperature ranges (Table 1) significantly decrease for the doped materials as compared to the pristine ZnO, likely due to the electron donor effect. Still, the difference in *E_a_* for the doped compositions is rather minor, except the case of the heavily doped *x* = 0.008 sample at 522–822 K, where additional effects can be imposed by the growing amount of ZnTa_2_O_6_ phase. 

A comparison between the electrical conductivity values of the doped samples suggests that initial good homogenization of the components may be a critical issue for the materials prepared via solid-state route, especially when the amount of dopant is small and the formation of dielectric phases like ZnTa_2_O_6_ [30,33] may be thermodynamically favoured instead of desirable electronic doping. A noticeable decrease of the electrical conductivity from *x* = 0.002 to *x* = 0.004 can be ascribed to the blocking effects at the grain boundaries, provided by the formation of ZnTa_2_O_6_ layers. The spatial distribution of these layers and their potential effect on the percolation of conducting wurtzite phase does not appear to be very different between *x* = 0.004, 0.006 and 0.008 samples, as suggested by the EDS maps shown in Figure 4D–F. Thus, although ZnTa_2_O_6_ impurities were already observed even for *x* = 0.002, further increase in tantalum content may still result in n-type doping and moderate conductivity increase in the sequence *x* = 0.004, 0.006 and 0.008. This is also confirmed by the overall decrease of the absolute values of the Seebeck coefficient from pristine ZnO to *x* = 0.008 (Figure 5B), indicating a progressive generation of the n-type charge carriers on *x* increase. The lowest |*α*| values are, thus, observed for heavily doped *x* = 0.008 material. As compared to other compositions, the electronic transport in this sample can be significantly affected by the dislocation scattering mechanisms, in accordance with the results of TEM characterization (Figure 2). In this case, the acceptor states are formed along the dislocation line and act as a trap for the n-type charge carriers [6]. Thus, although the results of the electrical characterization suggest that the *x* = 0.008 sample may possess the highest charge carrier concentration, the mobility of the charge carriers in this composition can be strongly affected by the dislocation scattering, leading to the relatively low electrical conductivity.

**Figure 6:**
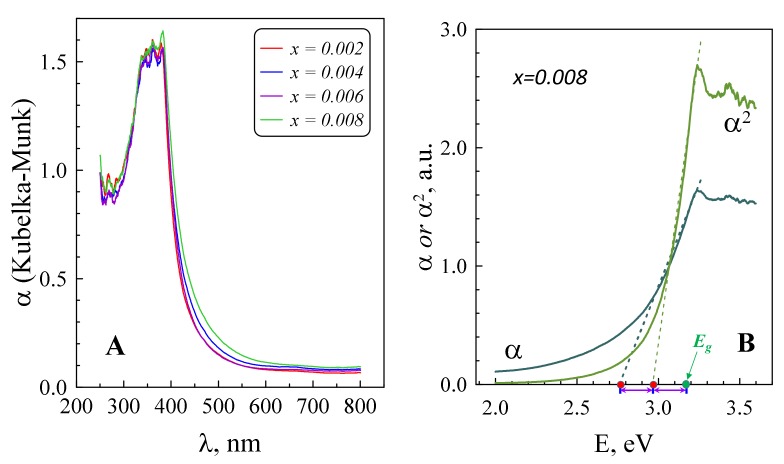
Diffuse reflectance spectroscopy (DRS) spectra of the prepared materials, represented through absorption coefficient *α* (Kubelka–Munk formalism) (**A**) and representative example for the band gap calculation by a method adopted in [34] (**B**). Corresponding DRS spectrum for pristine material can be found elsewhere [17].

The above discussion of the tantalum doping effects on the concentration of the charge carriers is further supported by the results of DRS characterization shown in Figure 6. 

The optical band gap for Ta-doped samples was calculated from DRS data (Figure 6A), involving an approach described in [34], which takes into account doping-induced electron-electron, electron-impurity scattering and Burstein–Moss effect by considering the difference in the extrapolated x-intercepts of the linear ranges of the *α* vs. *hν* and *α^2^* vs. *hν* graphs, as shown in Figure 6B. Significant widening of the *E_g_* from pristine ZnO to *x* = 0.002 composition can be attributed to the Burstein–Moss effect, when the electrons generated by doping block the low-energy transitions; similar behaviour was observed in [17]. Further band narrowing is often observed in heavily-doped ZnO [35,36] and ascribed to the many-body effects on the conduction and valence bands due to progressive generation of the charge carriers. It also should be mentioned that the *E_g_* value can be also affected by the presence of dislocations [37]; the combined effects might be responsible for the noticeable band gap decrease from *x* = 0.006 to 0.008.

### 3.3. Thermoelectric Performance

**Figure 7:**
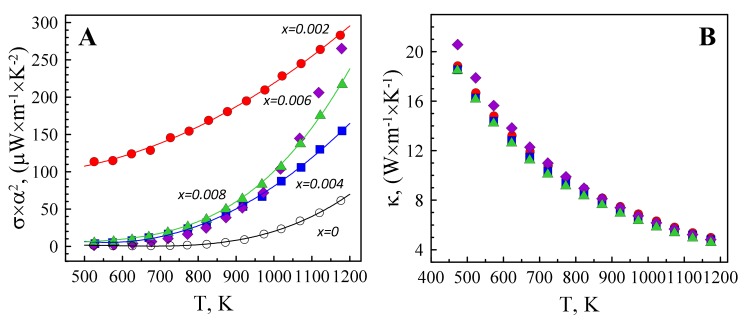
Temperature dependence of the power factor (**A**) and thermal conductivity (**B**).

The effects of Ta-doping on cumulative electrical performance of Zn_1−x_Ta_x_O, represented by the power factor, and thermal transport are shown in Figure 7. 

Despite relatively low electrical conductivity, hardly reaching 13–14 S/cm in the studied temperature range, the power factor for *x* = 0.002 sample is essentially high and comparable to that obtained for other ZnO-based thermoelectrics containing rather “traditional” dopants, including aluminium, and prepared via a solid-state route [9,16,17,19]. The latter is provided by a relatively high Seebeck coefficient of Zn_0.998_Ta_0.002_O, reaching −464 and −349 μV/K at 1175 and 830 K, accordingly, to result in the power factor of 0.28 and 0.17 mW × m^−1^ × K^−2^. At high temperatures, the differences in the *σ* × *α^2^* values between *x* = 0.002, 0.006 and 0.008 samples drastically decrease. By gaining an additional energy on heating, the charge carriers become less susceptible to trapping by the acceptor states, while the contribution of polar optical phonons to the carrier scattering increases for all samples [38]. 

**Figure 8:**
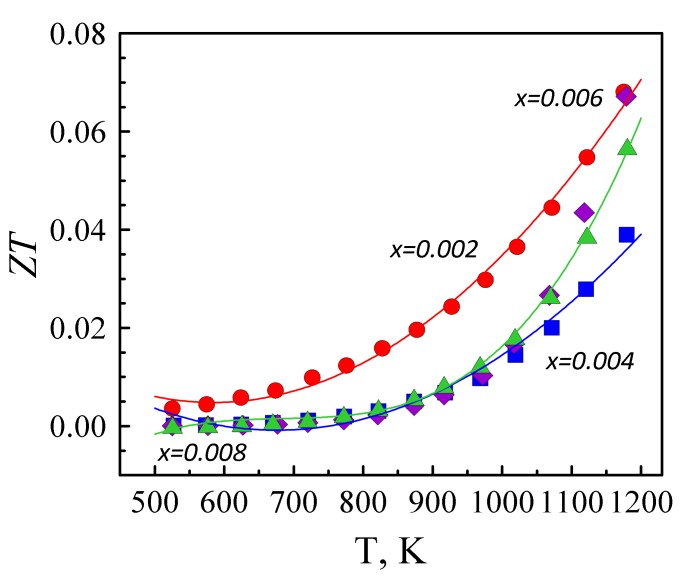
Temperature dependence of the dimensionless figure-of-merit.

Surprisingly, the thermal conductivity values are almost not affected by Ta doping and appearance of the phase impurity and dislocations. It should be noticed that the contribution of the lattice counterpart to the overall thermal transport exceeds 99% for all samples, as estimated from the Wiedemann–Franz´s law. The doping level is apparently too small to introduce a sufficient amount of the atomic defects for effective phonon scattering, while the size of ZnTa_2_O_6_ inclusions appears to be too large for that (Figure 4). Slightly lower thermal conductivity of the *x* = 0.008 sample may still result from the phonon scattering at dislocations [39]. Thus, the main contribution to the variation of thermoelectric figure-of-merit (Figure 8) with composition is provided by the electrical properties. 

The crossover of *ZT*´s for *x* = 0.002, 0.006 and 0.008 samples at high temperatures is apparently linked to the change of the charge carrier scattering mechanism, as mentioned above. The obtained *ZT* values are typical for donor-doped ZnO processed by the solid-state route, indicating that tantalum can be considered as a potential dopant for promoting the thermoelectric performance in zinc oxide, although its solubility in the wurtzite lattice is low. In general, the doping level can be enhanced by better homogenization of the initial precursors using wet chemical synthesis and combustion methods, and by relying on mutual solubility effects in the case of co-doping strategies [17,40,41].

## 4. Conclusions

In order to assess tantalum as a dopant to promote thermoelectric properties in zinc oxide, a series of the Zn_1-x_Ta_x_O (*x* = 0–0.008) samples was prepared by conventional solid-state route from oxide precursors, followed by sintering at 1773 K for 10 h in air. XRD analysis confirmed the presence of wurtzite as the main phase and showed significant variations of the *c* lattice parameter on doping, linked to the formation of highly defective structures. Combined XRD/HRTEM/SEM/EDS studies revealed a significant amount of dislocations in highly doped samples and the formation of ZnTa_2_O_6_ impurity, indicating that actual doping level may not exceed 0.002 at.%. The guidelines obtained from the diffuse reflectance spectroscopy and electrical studies suggested that the Ta doping level in nominal compositions Zn_1−x_Ta_x_O may still increase with *x*, while being accompanied with the formation of various defects and ZnTa_2_O_6_ phase at the grain boundaries with blocking effects on the electronic transport. The thermal conductivity of the prepared materials was found to be almost independent on tantalum content. The maximum thermoelectric performance was observed for the *x* = 0.002 sample and amounted to *ZT* of ~0.07 at 1175 K.

## Figures and Tables

**Table 1:** Selected properties of the samples with nominal composition Zn_1−x_Ta_x_O.

x	ρ_exp_/ρ_theor_ * (%)	Activation Energy of the Electronic Transport		*E_g_* ** (eV)
		Temperature Range (K)	*E_a_* (kJ/mol)	
0	91	873–1177 675–873	54 ± 2 48 ± 4	3.14 [17]
0.02	92	-	-	3.24
0.04	93	822–1180 573–822	18 ± 1 23 ± 1	3.21
0.06	93	820–1179 525–820	22 ± 1 23 ± 1	3.21
0.08	94	822–1180 522–822	22 ± 1 12 ± 2	3.17

* ratio between experimental and theoretical density; ** room-temperature optical band gap.
